# Enhancing tofacitinib’s therapeutic efficacy in murine arthritis with a synbiotic formulation comprising *Bacillus megaterium* DSM 32963 and an Omega-3 fatty acid lysine salt

**DOI:** 10.3389/fimmu.2025.1540878

**Published:** 2025-05-26

**Authors:** Annette Zehrer, Alexandra Rausch, Paul M. Jordan, Oliver Werz, Heike Tom Dieck, Thomas Berngruber

**Affiliations:** ^1^ Business Unit Microbiotica, Weber & Weber GmbH, Inning, Germany; ^2^ THR Cross Indication Research, NUVISAN ICB GmbH, Berlin, Germany; ^3^ Department of Pharmaceutical/Medicinal Chemistry, Institute of Pharmacy, Friedrich Schiller University Jena, Jena, Germany; ^4^ Creavis, Evonik Operations GmbH, Hanau, Germany

**Keywords:** probiotic, SPM, n3-PUFA, synbiotic, rheumatoid arthritis, nutritional supplement, adjuvant therapy, chronic inflammation

## Abstract

**Introduction:**

Omega-3 polyunsaturated fatty acids (n3-PUFA) are known for their anti-inflammatory benefits, particularly in chronic conditions like rheumatoid arthritis (RA). To resolve an acute inflammation, conversion of n3-PUFA into specialized pro-resolving mediators (SPM) is crucial. Recently, it was shown that the probiotic Bacillus megaterium DSM32963 supports this conversion.

**Methods:**

This study evaluates a synbiotic formulation combining Bacillus megaterium DSM32963 and a unique n3-PUFA-lysine salt as adjunct nutritional supplement to tofacitinib in adjuvant-induced arthritis (AIA) in rats.

**Results:**

Our findings reveal that a combination of low-dose tofacitinib and the synbiotic (ldTofa+Syn) significantly improved all measured arthritis severity parameters, outperforming either single treatment as well as supplementation with a conventional omega-3 ethyl ester that showed no effects on disease severity. The ldTofa+Syn combination also led to a notable reduction in C-reactive protein (CRP) and markers of NETosis in joint tissue, with a significant decrease in neutrophil chemokine CXCL1 observed only in synbiotic-containing groups. Additionally, there was a marked trend towards lower levels of the key inflammatory cytokines TNFα, IL-1β, and IL-6 in the ldTofa+Syn group.

**Conclusion:**

In conclusion, the specific synbiotic formulation shows promise as a complementary nutritional therapy for RA, improving disease outcomes and modulating immune responses.

## 1 Introduction

Chronic inflammation is the cause of numerous health concerns worldwide, including rheumatoid arthritis (RA), a chronic autoimmune-mediated inflammatory disorder characterized by synovitis and joint destruction (1, 2). Early diagnosis and therapeutic intervention are privotal in RA, as the initial months post-symptom onset present a unique window to alter the disease’s trajectory (3).

Omega-3 polyunsaturated fatty acids (n3-PUFA) have garnered attention for their role in inflammation regulation, with evidence linking them to positive arthritis outcomes (4–7).

The anti-inflammatory capabilities of n3-PUFA are attributed mainly to their metabolites, specialized pro-resolving mediators (SPM), produced by mammalian lipoxygenases (8, 9).

However, critics challenge this paradigm, noting that clinical studies have failed to consistently detect increased SPM plasma levels in humans after fish oil supplementation (10–12). Recent research suggests SPM can function as pro-resolving mediators by modulating the prostaglandin E2 receptor EP4 leaving room for discussion about the mechanisms behind n3-PUFAs’ anti-inflammatory effects (13). SPM are lipid mediators that are part of a larger family of pro-resolving molecules. The group of SPM includes maresins, protectins, resolvins, and lipoxins, along with their precursors (9). However, various physiological states, including RA, can impede SPM formation, thereby diminishing the effectiveness of n3-PUFA supplementation (14–16). RA patients often exhibit impaired SPM levels (17–20), prompting research into the therapeutic administration of SPM in experimental arthritis models (21–24).

Interestingly, it is also indicated that n3-PUFA are connected with the gut microbiota, as n3-PUFA can alter the composition of the microbiome, and improve the gut barrier integrity (25, 26). Conversely, the microbiota seems to affect the metabolism and absorption of n3-PUFA (27, 28). This interaction of n3-PUFA with the gut microbiome offers an opportunity to support the conversion of n3-PUFA to SPM via gut microbiome modulation. In previous work, we identified the natural and food-grade probiotic strain *Bacillus megaterium* (renamed to *Priestia megaterium*) DSM32963 that is able to convert n3-PUFA to SPM precursors via the bacterial oxygenase gene CYP102A1, independent of mammalian lipoxygenase activity (29). Recently, it was demonstrated that a synbiotic composition comprising this strain and n3-PUFA lysine salts was able to raise plasma levels of the SPM precursors 5-HEPE and 18-HEPE in healthy volunteers (30).

In the present study, we examined the impact of this synbiotic composition in a rat-specific formulation on arthritis. Utilizing the established adjuvant-induced arthritis (AIA) model, we compared the synbiotic strategy against treatment with the Janus kinase inhibitor tofacitinib and standard n3-PUFA supplementation. We also assessed the synbiotic’s role as a supplementary nutritional intervention alongside low-dose Tofacitinib, potentially allowing a dose reduction for disease-modifying anti-rheumatic drugs (DMARD)s. This reduction is particularly relevant given the increased risk of severe side effects associated with this potent Janus kinase inhibitor in certain patient groups (31) and the recommendation to reduce the dose in these patients (3).

Our results indicate that the synbiotic approach not only surpasses traditional n3-PUFA supplementation but also enhances the effectiveness of low-dose tofacitinib, significantly mitigating the severity and progression of arthritis.

## 2 Material and methods

### 2.1 Animals and experimental design

Fifty-two 7-week-old male Lewis rats (Ch. River, Germany) weighing ~200 g were housed in a barrier facility (12 h light/12 h dark cycle) accredited by the Association for Assessment and Accreditation of Laboratory Animal Care International (AAALAC) with free access to food and water *ad libitum*. Upon arrival, animals were randomly assigned to five treatment groups and two control groups, as follows (1): Healthy Ctrl.: healthy control rats (vehicle only); arthritic AIA rats administered with: (2) AIA Ctrl.: AIA control rats (vehicle only); (3) 6 mg/kg tofacitinib: rats treated with the human equivalent (5 mg twice/day) exposure of tofacitinib (LC Laboratories, 6 mg/kg), as determined by internal PK-/PD-studies on rats by NUVISAN ICB GmbH, based on Dowty and colleagues (32); (4) 3 mg/kg tofacitinib: rats treated with a low-dose of the targeted synthetic (ts) DMARD; (5) Synbiotic: rats receiving the synbiotic consisting of a particular lysine salt of the n3-PUFA EPA and DHA and *Bacillus megaterium* (73 mg/rat AvailOm^®^ supplemented with 2 x 10^8^ cfu *Bacillus megaterium* DSM32963 [B4U^®^63]; Evonik Operations GmbH); (6) ldTofa+Syn: rats treated with the combination of tofacitinib (3 mg/kg) and the synbiotic (73 mg/rat); (7) n3-PUFA: rats treated with a conventional omega-3 product containing omega 3-fatty acid ethyl ester (Omacor^®^, Mylan Healthcare, 44 µL/rat). These 44 µL correspond to the recommended human daily dose of 2 g/day, with 6-fold dose conversion for the rat model according to Nair and colleagues (33), and contain the same sum amount of EPA+DHA as in 73 mg of the synbiotic. Each group consisted of eight rats, except healthy controls with n = 4. Treatments were given once daily (QD) by oral gavage (po) starting with the disease induction (day 0) and continuing to day 15, one day before the rats were sacrificed.

### 2.2 Induction of rat adjuvant-induced arthritis

The rat AIA model was performed as described previously (34). Briefly, rats were injected subcutaneously at the tail base on day 0 with a single dose of 0.1 mL (10 mg/mL) of heat-killed *Mycobacterium tuberculosis* (strain H37Ra; Difco, USA) suspended in incomplete Freund´s adjuvant (IFA; Difco, USA) to induce adjuvant arthritis. The healthy, non-arthritic control group was not injected with the adjuvant. At the end of the study (day 16), the animals were anaesthetized by inhalation of isoflurane (induction of anesthesia: 5% isoflurane; maintenance of anesthesia: 2-3% isoflurane, with a constant flow rate of 1000mL/min O_2_ in both cases), blood samples from each animal were collected with sodium citrate as anticoagulant by bleeding via the vena cava, followed by cervical dislocation. Plasma was collected after centrifugation and frozen at –80°C. Ankle joints were harvested by transection of the hind paws at the distal tibia, proximal to the tarsal joint.

### 2.3 Clinical disease scores

Rats were monitored daily by assigning an arthritis disease activity score for each rat to macroscopically assess the disease onset and progression, as described by Baharav and colleagues (35). Each hind paw was scored on a 0–4 scale with 0 = normal; 1 = erythema and mild swelling confined to the midfoot (tarsals) or ankle joint; 2 = erythema and mild swelling extending from the ankle to the midfoot; 3 = erythema and moderate swelling extending from the ankle to the metatarsal joint; and 4 = erythema and severe swelling of the ankle, foot, and digit. The disease activity score was defined as the sum of the scores of both hind paws of each rat. Additionally, the extent of joint swelling was analyzed once before disease induction and starting from day 8, three times weekly by estimating the ankle cross-section area using sagittal and transversal measurements with an automatized caliper, as well as by determining the hind paw volume using a plethysmometer (IITC Life Science Inc., USA). In parallel, grip strength was analyzed as a marker for functional disability in each individual rat using a grip strength meter (IITC Life Sciences Inc., USA).

### 2.4 Bone mineral density

For the measurement of bone mineral density (BMD) of the right tarsal joint, animals were anaesthetized by inhalation of isoflurane as described above and BDM was determined via µCT imaging. For this purpose, the µCT MILabs U-CT/OI/FLT (MILabs, Netherlands) was used in the “normal” and “full” scan mode. Images were acquired at 50 kV, and the total acquisition time was 4 min, producing a 0.04 mm voxel image. Analysis was performed by standardizing images to QRM-MicroCT-HA phantom (QRM Moehrendorf, Germany) and using the software Imalytics-Preclinical (Gremse-IT, Germany). The used threshold for segmentation was 1200.

### 2.5 Histopathology

Right tarsal joints were fixed in 10% neutral buffered formalin. After decalcification, tarsal joints were cut paramedianly in the sagittal plane. After routine dehydration, the samples were embedded in paraffin, and 5 µm sections were stained with hematoxylin-eosin (H&E) for microscopic examination. The histopathological investigation was performed in a blinded fashion by a pathologist using a modified Knoerzer score (36). Scores from 0 to 3, with 0 = healthy, 1 = mild; 2 = moderate, 3 = severe, were given for each joint with respect to the degree of synovial hyperplasia, polymorphonuclear infiltration, mononuclear infiltration, periarticular inflammation, vasculitis, pannus formation, chondral erosions, subchondral bone lesions, periosteal proliferation and granulomatous nodules. The histopathological score was defined as the sum of the single parameter scores.

### 2.6 White blood cell count and erythrocyte sedimentation rate

Drawn whole blood from rats was evaluated for leukocyte counts after lysis of erythrocyte for 10 min at 37°C in ice-cold lysis buffer (BD Bioscience, Germany), and staining with propidium iodide (BD Bioscience, Germany). Cell counts for a fixed amount of sample volume were acquired using a flow cytometer (BD FACSCanto II; BD FACSDiva software, BD Bioscience, Germany).

The level of ESR as an indicative of inflammation was determined using the Westergren method. The tubes (BSG-Microvette CB 200 NC, Sarstedt, Germany) were mounted in a vertical position and ESR was read 2 h later as mm of clear plasma.

### 2.7 Processing of hind paws for *ex vivo* analysis

Left tarsal joints were immediately frozen in liquid nitrogen after removal. Frozen limbs were cyro milled (Retsch, Germany) and stored at -80°C until used. 150 mg of joint powder per rat was dissolved in 1 mL medium (RPMI-1640, Gibco, Germany) supplemented with complete mini ethylenediaminetetraacetic acid-free protease inhibitor cocktail (Roche, Germany) for cytokine and C-reactive protein (CRP) analysis or in 2 mL ice-cold homogenate buffer containing 0.5% hexadecyltrimethylammonium bromide and 10 mM 3-(N-morpholino)propanesulfonic acid (MOPS; Sigma Aldrich, Germany) (pH 7.0) for myeloperoxidase (MPO)- and neutrophil elastase activity determination.

### 2.8 Cytokine and CRP measurement by ELISA

Cytokine levels in joint homogenates and plasma were determined using commercially available multiplex ELISA that specifically recognize the rat cytokines TNFα, IL-1β, IL-6 and CXCL1 (Meso Scale Discovery, USA). CRP was measured using a CRP ELISA kit (BD Biosciences, USA). ELISAs were performed according to the manufacturer’s instructions.

### 2.9 Determination of neutrophil elastase and MPO activity in joint homogenates

Neutrophil elastase (NE) activity was measured by fluorescence of 7-amino-4-methyl-coumarin (AMC) that is released from the substrate MeO-Succ-Ala-Ala-Pro-Val-AMC (Bachem, USA) (37). The assay protocol was modified from Schottelius and colleagues by implementing a kinetic measurement (38). The substrate (300 mM in DMSO) was diluted 1:300 in 1 mM Tris-BSA buffer (pH 8.5). Thereafter, 25 µL of substrate was added to 25 µL of dissolved joint samples in a 96-microtiter plate and placed in the pre-warmed (37°C) plate reader (Spectra Max; Molecular Devices, USA) to start the kinetic measurement (λ Excitation = 380 nm, λ Emission = 460 nm, measurement every 30 sec) for a duration of 10 min. The determined Vmax (maximal initial velocity) was extrapolated via the AMC standard curve.

To measure MPO activity, tetramethylbenzidine (TMB) dihydrochloride was used as a sensitive chromogen substrate for peroxidase, as described previously (38). To convert TMB into TMB dihydrochloride, 34 µL of 3.7% hydrochloric acid (equimolar) was added to 5 mg of TMB. Then, 1 mL of DMSO was added. This stock solution was slowly added to sodium acetate-citric acid buffer (0.1 mol/L, pH 6.0) in a ratio of 1:100. 200 µL of this TMB solution, 40 µL of the homogenized sample, and 25 µL of 1 mM H_2_O_2_ were added to a microtiter plate to start the reaction. The reaction was stopped after 30 min with 45 µL of 1 N H_2_SO_4_. The extinction was detected at λ = 450 nm using a microtiter plate reader (Molecular Devices, USA) and extrapolated via the MPO standard curve.

Both enzymatic activities (MPO and NE) were referred to protein levels in joint tissue, which were determined by a commercially available kit beforehand (Pierce BCA Protein Assay-Kit, ThermoFisher Scientific, GermanyPierce) according to the manufacturer´s instructions.

### 2.10 Analysis of lipid mediators in plasma

For lipid mediator (LM) analysis using ultra-performance liquid chromatography-tandem mass spectrometry (UPLC-MS-MS) samples were transferred to 2 mL of ice-cold methanol containing 10 μL of deuterium-labeled internal standards (200 nM d8-5S-HETE, d4-LTB_4_, d5-LXA_4_, d5-RvD2, d4-PGE_2_, and 10 μM d8-AA; Cayman Chemical/Biomol GmbH, Hamburg, Germany) to facilitate quantification and sample recovery. Samples were then kept at -20°C for at least 60 min to allow protein precipitation. The extraction of LM was performed as recently published (39). In brief, after centrifugation (1200 × g; 4°C; 10 min) acidified H_2_O (9 mL; final pH = 3.5) was added and samples were extracted on solid phase cartridges (Sep-Pak^®^ Vac 6cc 500 mg/6 mL C18; Waters, Milford, MA, USA). Samples were loaded on the cartridges after equilibration with methanol followed by H_2_O. After washing with H_2_O and *n*-hexane, samples were eluted with methyl formate (6 mL). The solvent was fully evaporated using an evaporation system (TurboVap LV, Biotage, Uppsala, Sweden) and the residue was resuspended in 200 µL methanol/water (1:1, v/v) for UPLC-MS-MS analysis. LM were analyzed with an Acquity™ UPLC system (Waters, Milford, MA, USA) and a QTRAP 5500 Mass Spectrometer (ABSciex, Darmstadt, Germany) equipped with a Turbo V™ Source and electrospray ionization. LM were eluted using an ACQUITY UPLC^®^ BEH C18 column (1.7 µm, 2.1 mm × 100 mm; Waters, Eschborn, Germany) heated at 50°C with a flow rate of 0.3 mL/min and a mobile phase consisting of methanol-water-acetic acid at a ratio of 42:58:0.01 (v/v/v) that was ramped to 86:14:0.01 (v/v/v) over 12.5 min and then to 98:2:0.01 (v/v/v) for 3 min. The QTRAP 5500 was run in negative ionization mode using scheduled multiple reaction monitoring (MRM) coupled with information-dependent acquisition. The scheduled MRM window was 60 s, optimized LM parameters were adopted, with a curtain gas pressure of 35 psi. The retention time and at least six diagnostic ions for each LM were confirmed by means of an external standard for each and every LM (Cayman Chemical/Biomol GmbH). Quantification was achieved by calibration curves for each LM. Linear calibration curves were obtained for each LM and gave r^2^ values of 0.998 or higher. The limit of detection for each targeted LM was determined as described (39).

### 2.11 Statistical analysis

Data shown represent means ± 95% confidence interval (CI). Statistical significance was determined by one-way ANOVA, Welch-ANOVA (for data with unequal standard deviation), or two-way ANOVA with Dunnett *post hoc* test for multiple comparison against the AIA group treated with vehicle only using GraphPad Prism 9 (GraphPad Software). In case of additional testing against the group 3 mg/kg tofacitinib plus synbiotic, or against conventional omega-3 product, p values were adjusted using the Bonferroni method. A p value < 0.05 was considered significant.

## 3 Results

### 3.1 Adjuvant synbiotic treatment significantly improved effects of low-dose tofacitinib on disease progression

To investigate potential beneficial effects of the synbiotic composition on the progression of arthritis, rats were challenged with complete Freund’s adjuvant (CFA) and grouped to specific treatments: AIA Ctrl., 6 mg/kg tofacitinib, 3 mg/kg tofacitinib, synbiotic, ldTofa+Syn, and n3-PUFA. Besides AIA Ctrl., healthy Ctrl. rats served as controls.

**Figure 1:**
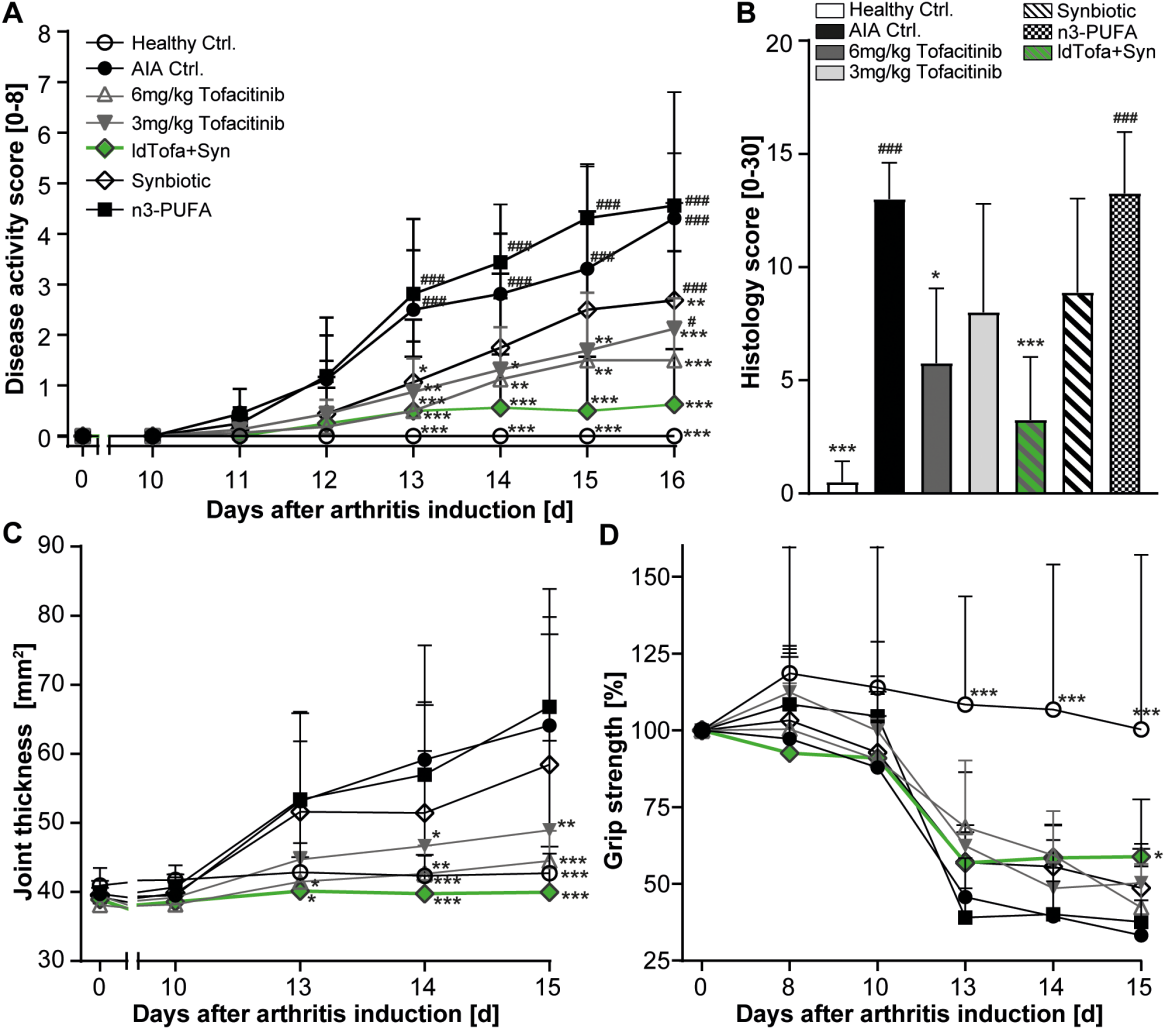
Superior reduction of arthritis severity by the combination treatment (ldTofa+Syn). **(A)** Development of disease activity score over time. **(B)** Histology score at endpoint. **(C)** Development of ankle cross-section area (sagittal x transversal) over time **(D)** Development of grip strength over time. Grip strength was normalized for each animal to measured values with a grip strength meter at day 0 (100%). n = 8/n = 4 healthy Ctrl.; Data represent mean +95% CI; *p < 0.05, **p < 0.01, ***p < 0.001 vs. AIA Ctrl.; additionally, tested for **(A, B)**: #p < 0.05, ###p < 0.001 vs. ldTofa+Syn.

Significant differences in arthritis severity appeared as early as day 13. Disease activity was evaluated by scoring both hind paws of each animal in relation to erythema and the extent of joint swelling (scores ranging from 0 = normal to 4 = severe). By day 16, the rats treated with both tofacitinib doses demonstrated a significantly reduced disease activity score compared to AIA Ctrl., as expected. The administration of the synbiotic or ldTofa+Syn also led to a significantly reduced arthritis score compared to AIA Ctrl., with ldTofa+Syn demonstrating superiority over the single treatments with either the synbiotic or 3 mg/kg tofacitinib alone (**Figure 1A**). The treatment with conventional n3-PUFA did not have a positive effect on disease activity in this study.

Histopathological examination of the animals’ left tarsal joint confirmed these findings. Based on ten relevant parameters (rated from 0 = no lesion to 3 = severe), the histology score showed a significant reduction for 6 mg/kg tofacitinib and ldTofa+Syn compared to AIA Ctrl. 3 mg/kg tofacitinib and the synbiotic alone demonstrated a clear trend toward reduction, whereas no effect was observed for n3-PUFA (**Figure 1B**).

Measurement of ankle cross-section area (**Figure 1C**) and paw volume (**Supplementary Figure S1A**) affirmed that ldTofa+Syn had the most beneficial effect on joint thickness. The high-dose of tofacitinib (6 mg/kg) seemed similarly effective.

Tracking of the development of grip strength over time revealed that only the daily administration of ldTofa+Syn attenuated the illness-related reduction of this parameter, which correlates with muscle strength and hyperalgesia. By day 15, the grip strength in the ldTofa+Syn group was significantly higher compared to the AIA Ctrl. group (**Figure 1D**).

Joint bone and cartilage destruction is a significant consequence of the inflammatory processes in RA. To evaluate the impact of different treatments on this parameter, we analyzed the BMD of the hind paws’ total joint using µCT imaging on day 16. As expected, the AIA Ctrl. animals displayed severe bone destruction. However, we did not observe an improvement in BMD of the total joint with any of the administered treatments in this study (**Supplementary Figure S1B**). Exploratory analysis of the BMD of the calcaneus, the most affected bone in this model, revealed a small trend to preserve BMD for the treatment with 6 mg/kg tofacitinib and ldTofa+Syn (**Supplementary Figure S1C**).

To better assess the physical condition of the animals during the study, and to be able to allow timely identification of any profound weight loss, the animals were weighed daily. The weight loss in the AIA Ctrl. and the treatment groups were as expected and did not reach the critical reduction of -20% for any of the animals (**Supplementary Figure S1D**). It’s worth noting that the weight of animals in the ldTofa+Syn group was slightly but consistently lower than the weight of animals in the other treatment groups.

### 3.2 Effect of treatments on markers of inflammation

At the end of this study, on day 16, the animals were sacrificed, and blood and joints were harvested for further analysis.

#### 3.3.1 Alteration of selected lipid mediators in the plasma

By employment of targeted UPLC-MS-MS-based metabololipidomics, we examined the levels of PUFA, namely, eicosapentaenoic acid (EPA), docosahexaenoic acid (DHA), and arachidonic acid (AA), as well as their specific metabolites, which play crucial roles in driving or mediating the inflammatory processes in RA (40). Our investigation focused on the following treatment groups: AIA Ctrl., synbiotic, ldTofa+Syn, and n3-PUFA.

**Figure 2:**
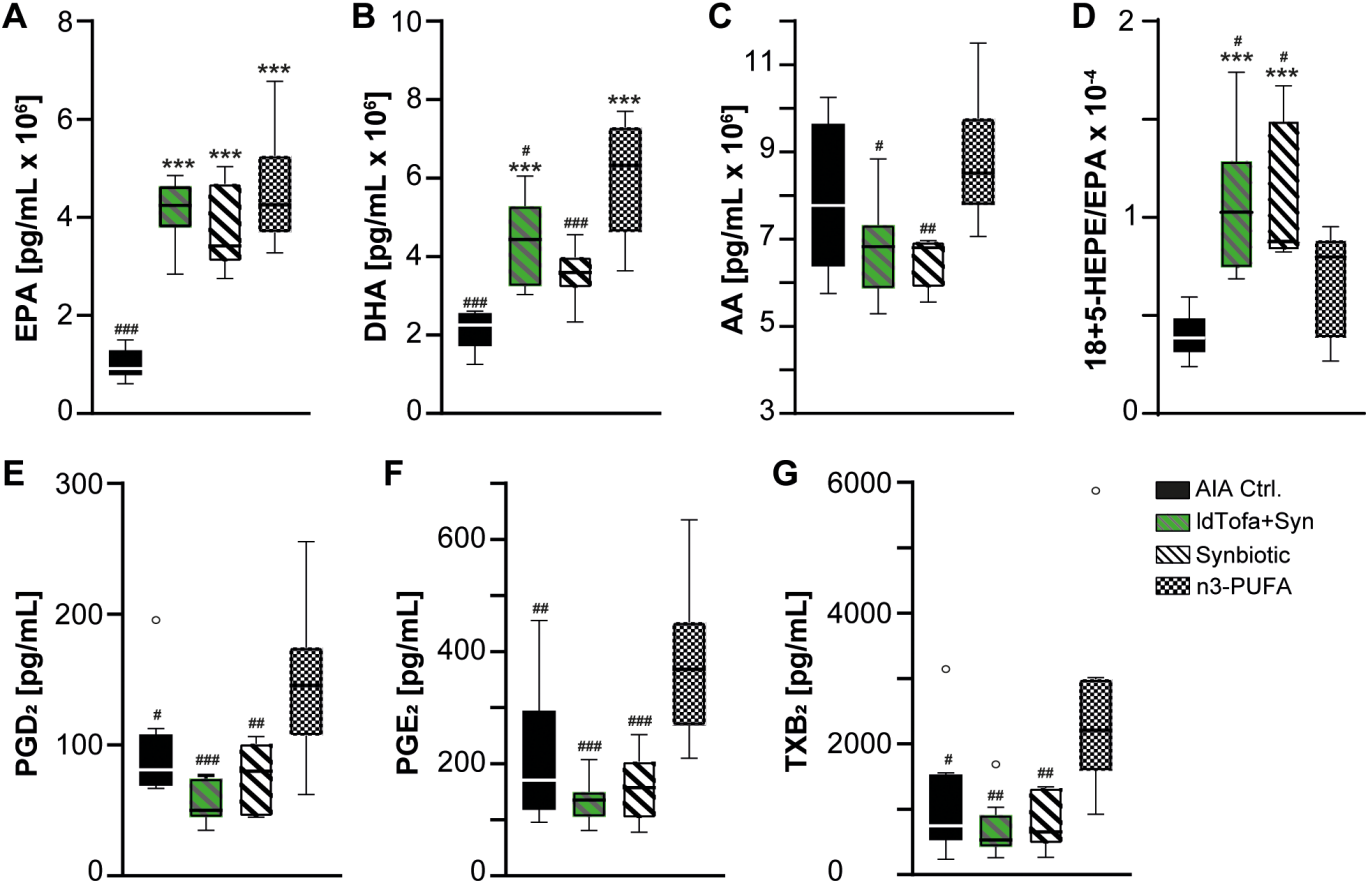
Effects of treatments on lipid mediator levels in plasma. **(A)** EPA **(B)** DHA, **(C)** AA, **(D)** Ratio 18-HEPE+5-HEPE/EPA, **(E)** PGD2, **(F)** PGE2, **(G)** TXB2, n = 8; Boxplot data represent median, 25th and 75th percentiles, whiskers drawn within the 1.5 IQR value (Tukey method plot), and outliers (empty circles). ***p < 0.001 vs. AIA Ctrl.; #p < 0.05, ##p < 0.01, ###p < 0.001 vs. n3-PUFA.

The analysis revealed significantly increased levels for EPA and DHA for synbiotic, ldTofa+Syn, n3-PUFA compared to AIA Ctrl. (**Figures 2A, B**). Notably, the main source of AA was the lard in the rodents’ standard pellet diet, resulting in high blood levels across all groups. Interestingly, the data demonstrated a significantly reduced amount of AA and DHA in the blood of animals that received the synbiotic compared to n3-PUFA (**Figures 2B, C**), suggesting that the synbiotic could promote the metabolization of AA and DHA.

A special focus of LM analysis was laid on the sum of the EPA-derived 18-HEPE and 5-HEPE, as in a previous study, this sum was significantly elevated in heathy volunteers after supplementation with the synbiotic (30). 18-HEPE and 5-HEPE are precursors of RvE1 and RvE2, 18-HEPE is the precursor of RvE3, and 5-HEPE is one of the precursors of RvE4 (41–43). To assess how effective EPA is metabolized towards RvE in the different groups, we calculated the ratio of 18-HEPE + 5-HEPE to EPA for each animal. The levels of 18 + 5-HEPE were significantly increased in all 3 treatment groups compared to AIA Ctrl., but seem to be similar between the treatment groups (**Supplementary Figure S2A**). However, the ratio of 18 + 5-HEPE/EPA, demonstrated a significantly increased ratio for the groups receiving synbiotic and ldTofa+Syn in comparison to n3-PUFA and AIA Ctrl. (**Figure 2D**), suggesting a more effective metabolization in the presence of the synbiotic. Notably, di- and trihydroxylated SPM could not be detected in the rat plasma with our system.

Furthermore, the data revealed a significant reduction of three pro-inflammatory AA-derived cyclooxygenase products. In detail, prostaglandin (PG)D_2_, PGE_2_ and thromboxane (TX)B_2_ were significantly reduced in the synbiotic group compared to the n3-PUFA group, which appears even more prominent in the ldTofa+Syn group (**Figures 2E-G**).

#### 3.3.2 Blood parameters

After the 16-day intervention, we analyzed several parameters related to inflammation in the blood of the rats.

Erythrocyte sedimentation (ESR) was assessed as a marker for unspecific systemic inflammation. The data demonstrated only a trend for elevated ESR levels in AIA Ctrl. animals compared to healthy Ctrl. with pronounced individual variations. None of the treatments seemed to effectively reduce ESR (**Supplementary Figure S2B**).

White Blood Cell Count (WBC) served as a marker for leukocytosis, which can be associated with more active arthritis in this short-term model. As expected, animals in the AIA Ctrl. group had significantly increased WBC compared to healthy Ctrl. Both tofacitinib treatments had no effect on this parameter. However, the synbiotic, n3-PUFA, and especially the ldTofa+Syn group demonstrated a trend towards reduced WBC, with the WBC in the ldTofa+Syn group being significantly lower than in the group receiving 3 mg/kg tofacitinib alone (**Supplementary Figure S2C**).

We evaluated the concentration of IL-6 in the blood of the animals, as this cytokine is strongly expressed and released in RA patients and its concentration was shown to correlate with disease activity (44). As expected, we detected a high and significant upregulation of IL-6 in the blood of AIA Ctrl. animals compared to healthy Ctrl. However, the different treatments in this study did not significantly affect the concentration of IL-6 in the blood (**Supplementary Figure S2D**).

#### 3.3.3 C-reactive protein in joints

**Figure 3:**
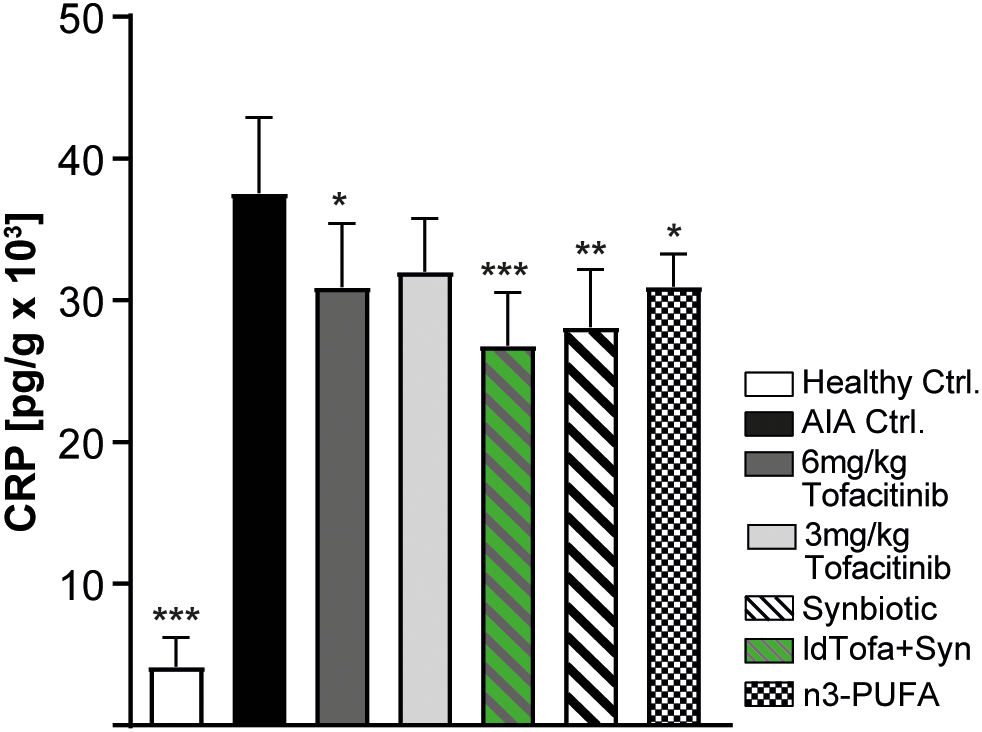
Effects of treatments on C-reactive protein (CRP) in joints. n = 8/n = 3 healthy Ctrl. Data represent mean +95% CI; *p < 0.05, **p < 0.01, ***p < 0.001 *vs*. AIA Ctrl.

A significant and widely used marker for general inflammation is the acute-phase serum protein CRP. Current data suggest that in patients with RA it is locally produced in the synovial tissues by fibroblast-like synoviocytes (45). In this study, we found as expected that AIA Ctrl. rats had highly increased CRP levels in their joint tissue compared to healthy Ctrl. Treatment with 6 mg/kg tofacitinib led to a small but significant decrease and low-dose tofacitinib treatment showed a trend in that direction. CRP levels decreased substantially in combination with the synbiotic (ldTofa+Syn). Even alone, the synbiotic decreased CRP levels significantly and seemingly more than the tofacitinib treatments. Treatment with n3-PUFA also resulted in a small but significant reduction of CRP in the joints compared to AIA Ctrl. (**Figure 3**).

#### 3.3.4 Cytokine production in the joints

**Figure 4:**
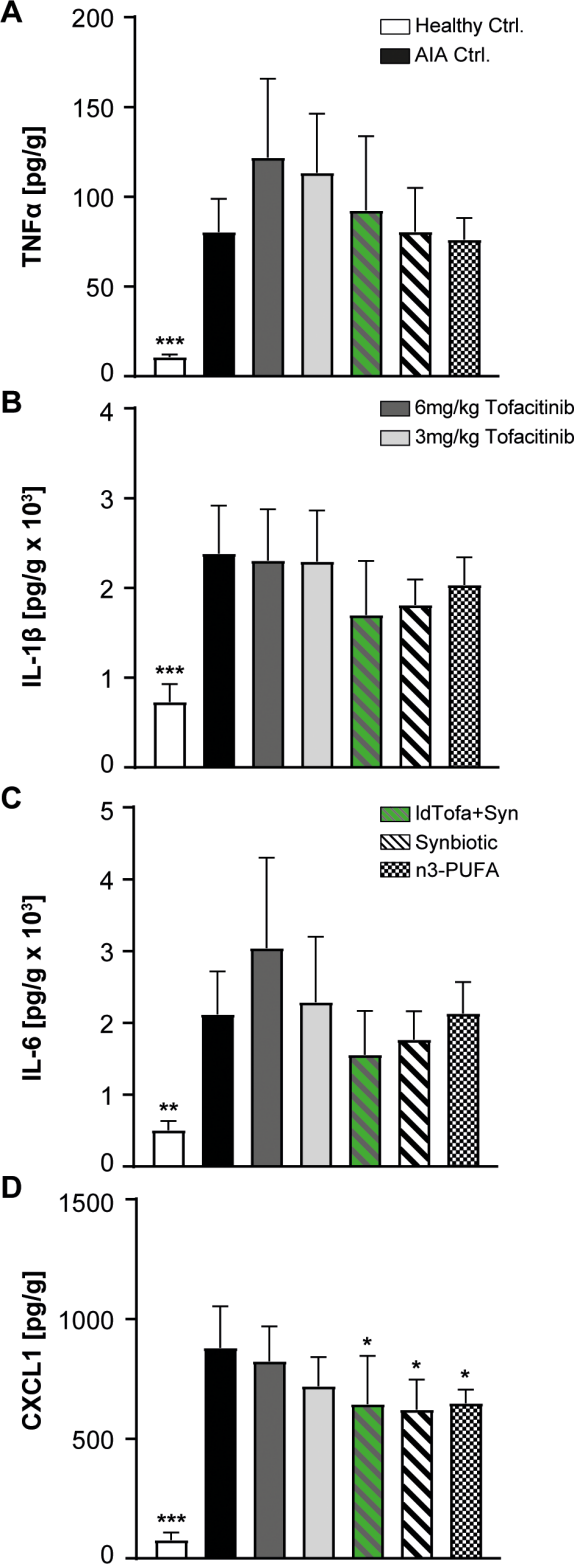
Effects of treatments on levels of pro-inflammatory and neutrophil attracting cytokines in joint tissue. Cytokine levels measured with ELISA for **(A)** TNFα **(B)** IL-1β **(C)** IL-6 **(D)** CXCL1. n = 8/n = 3 for healthy Ctrl.; Data represent mean +95% CI; *p < 0.05, **p < 0.01, ***p < 0.001 *vs*. AIA Ctrl.

IL-6, IL-1β and TNFα are important cytokines in the pathogenesis and progression of RA. In the joints, IL-6 was shown to promote enhanced endothelial production of the neutrophil chemoattractant CXCL1 (44). In this study, tofacitinib treatment had no or only minor effects on the protein levels of TNFα, IL-6, IL-1β, and CXCL1 in the joints (**Figures 4A-D**). In the treatment groups, the lowest value for TNFα was detected for the synbiotic and n3-PUFA, whereas the treatment with ldTofa+Syn resulted in TNFα levels between the synbiotic and 3 mg/kg tofacitinib (**Figure 4A**). The evaluation of the concentration of IL-1β revealed a distinct but not statistically significant reduction compared to the AIA Ctrl. for the synbiotic and the ldTofa+Syn group, whereas the treatment with n3-PUFA seemed to have minor effects (**Figure 4B**). IL-6 expression was noticeably reduced only in animals treated with synbiotic or ldTofa+Syn (**Figure 4C**). The protein amount of CXCL1 in the joints was significantly reduced compared to AIA Ctrl. in the groups treated n3-PUFA, the synbiotic and ldTofa+Syn (**Figure 4D**).

#### 3.3.5 Polymorphonuclear leukocyte infiltration and NETosis in joints

SPM play a crucial role in modulating the inflammatory response and were shown to possess anti-inflammatory effects on polymorphonuclear leukocytes (PMN), including decreased cell activation, migration and adhesion, reduced reactive oxygen species (ROS) generation, and inhibition of neutrophil extracellular traps (NETs) formation (9).

**Figure 5:**
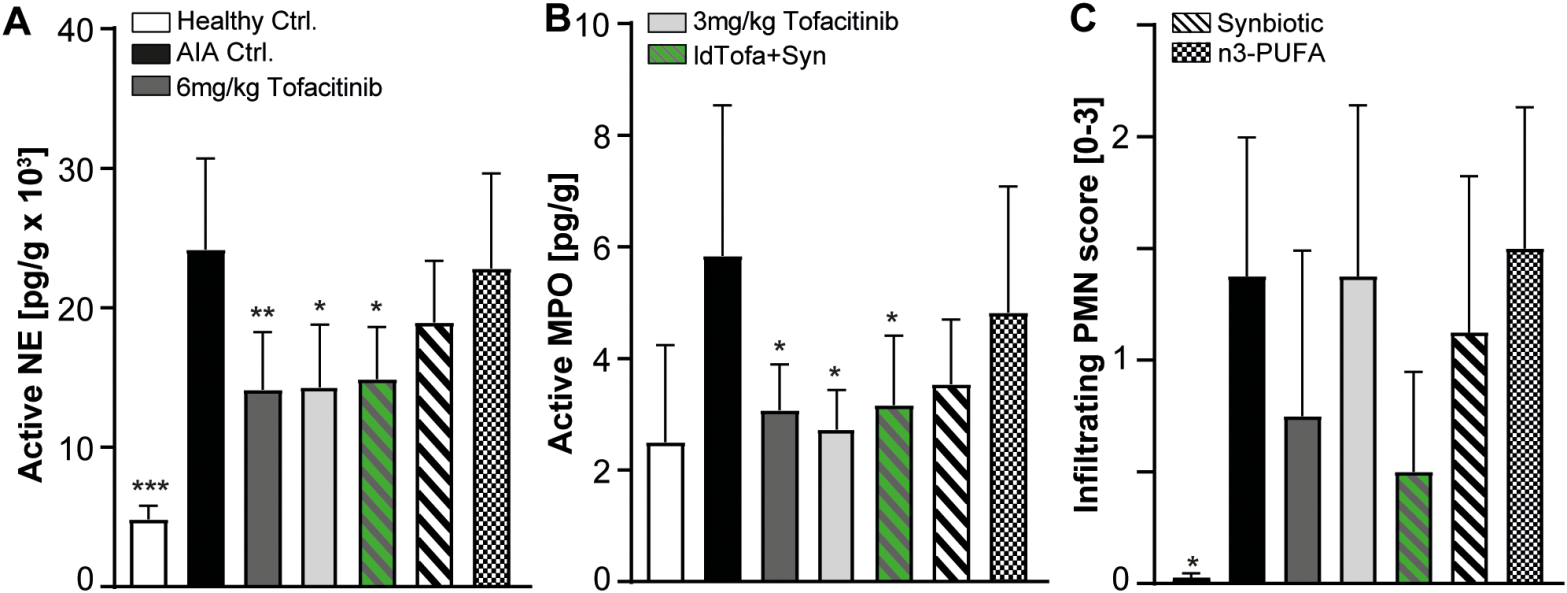
Effects of treatments on Myeloperoxidase (MPO), Neutrophil Elastase (NE) and infiltrating PMN in joints. **(A)** NE activity, **(B)** MPO activity, **(C)** Infiltrating PMN score. n = 8/n = 3 healthy Ctrl.; Data represent mean +95% CI; *p < 0.05, **p < 0.01, ***p < 0.001 *vs*. AIA Ctrl.

We analyzed the levels of active neutrophil elastase (NE) and myeloperoxidase (MPO) as parameters for NETosis in the joint homogenates of all experimental groups using specific activity assays (46). Additionally, we histologically evaluated PMN infiltration by assessing a sub-score of the histopathology score. As expected, both, MPO and NE activity was significantly reduced with tofacitinib compared to AIA Ctrl. Interestingly, the human equivalent exposure seemed to have no benefit over the low-dose. For the synbiotic alone, a clear trend for reduction in MPO and NE was shown; however, significance was reached only in combination with low-dose tofacitinib (**Figures 5A, B**). N3-PUFA treatment had no or only minor effects on the level of NETosis in this model.

PMN infiltration into the joints was significantly increased in the AIA Ctrl. group compared to healthy Ctrl., as expected. The treatment with n3-PUFA seemed to have no effect on the number of PMN in the joints, whereas 6 mg/kg tofacitinib and ldTofa+Syn demonstrated a strong trend to diminished PMN infiltration. In contrast, low-dose tofacitinib and the synbiotic alone did not substantially influence PMN numbers in the joints (**Figure 5C**).

In summary, our results demonstrate a beneficial impact of the nutritional synbiotic intervention alone, and especially on top of low-dose tofacitinib, on arthritis severity and progression as well as markers of inflammation.

## 4 Discussion

Medical guidelines recommend lifestyle improvements such as a change in diet for RA patients. For the nutritional supplementation with n3-PUFA, some more extensive studies point to a potential benefit if applied in high-doses (5, 7, 47). For patients, these high-doses are impractical to implement in their day-to-day lives and recent studies suggest that higher concentrations of esterified n3-PUFA could bear some risks (48). A further challenge with n3-PUFA supplementation is that RA patients often show disrupted levels of n3-PUFA-derived SPM (18, 19). To bypass these challenges, we used a selected bacterial strain to support SPM production by targeted metabolization of a distinct n3-PUFA-lysine salt preparation. In contrast to conventional, biologic or tsDMARDs that suppress the immune reaction, this approach aims at promoting inflammation resolution.

### 4.1 Model of arthritis

We chose an acute model of arthritis to investigate the impact of the synbiotic approach, as the critical phase for RA treatment is early in disease progression (3, 49). The supplementation regime was started in parallel to the induction of RA and not at the peak of disease, as nutritional interventions are likely to have the biggest impact in the early stages of the disease (50). Because our study’s aim was to focus on moderately severe arthritis, the experiment was terminated on day 16, before the onset of severe to very severe disease.

The AIA model used in this study is one of the most widely applied models for preclinical testing of new drugs for arthritis (51–55). It has a reliable incidence rate, essential features of human arthritis such as swelling of the extremities, synovial hyperplasia, lymphocyte infiltration into the joints, cartilage degradation and bone loss leading to loss of joint function (52, 56, 57), and is able to reflect key essential cytokines such as TNFα, IL-6, IL-1β known to be involved in the pathogenesis of human arthritis (58, 59). Nevertheless, this model of arthritis does not perfectly represent the disease situation in human patients, since the chronic progression in humans requires constant monitoring of disease activity over the years and an adaption of the treatment regime in case remission or low disease activity is not reached or maintained.

### 4.2 Arthritis severity and progression

In line with previous studies using this model, symptoms of the disease started from day 11 and continuously aggravated over time, resulting in significantly reduced grip strength, swollen joints and increased disease activity scores (52, 53). To evaluate the effect sizes of our synbiotic treatment and to precisely asses the influence of our new approach on relevant disease parameters, we analyzed the synbiotic in comparison to and in combination with the potent tsDMARD tofacitinib.

As expected, tofacitinib was able to alleviate the symptoms in a dose-dependent manner (32, 60), but in contrast to previous studies using n3-PUFA supplementation, the human-equivalent daily dose of n3-PUFA used in this study had no effect on disease progression (61–64). Different study designs and RA models, dissimilar n3-PUFA products, and differences in n3-PUFA dosages could be reasons for this discrepancy.

We speculate that the significant positive effect of the synbiotic on disease progression could be due to the known capacity of the bacterium to promote the metabolization of n3-PUFA towards anti-inflammatory metabolites such as 18-HEPE and 5-HEPE, and may be sustained by yet unknown mechanisms, as previous studies demonstrate that some probiotic bacteria positively affect arthritis severity (49, 65, 66).

In the present study, ldTofa+Syn was the superior treatment for all disease severity parameters, was significantly more efficient than both treatments alone regarding the disease activity score, and was the only treatment reaching significance against AIA Ctrl. for grip strength. These findings point towards a synergistic or complementary effect of low-dose tofacitinib and the synbiotic.

In this study, the BMD analysis via µCT imaging revealed positive trends only for 6 mg/kg tofacitinib and ldTofa+Syn. This finding is in line with other studies, demonstrating an effect only for higher doses of tofacitinib (60) or no effect of tofacitinib, despite an attenuated bone erosion score seen in histology (67). This suggests, that tofacitinib may not influence structural properties of the bones such as mineral density. In the clinic, bone erosion is mostly assessed using radiography, but data on bone fragility is scarce (68, 69), with one study showing that one-year tofacitinib therapy stabilizes bone mineral density in RA patients (70). Possibly, a positive impact of tofacitinib needs longer periods of time to manifest, which could not be depicted in the present study. To our knowledge, human data on the effect of n3-PUFA or probiotic bacteria on bone mineral density in RA is still missing, but a few rodent models report a positive effect on micro-CT scores and bone mineral content (49, 71).

### 4.3 Lipid mediators

N3-PUFA-derived SPM are LM that are important mediators for resolution of inflammation and can modulate the levels of important markers of inflammation (17). We limited the analysis to four groups, namely ldTofa+Syn, synbiotic, n3-PUFA, and AIA Ctrl.

Our data for the n3-PUFA EPA and DHA clearly indicate that the supplementation of the animals was successful, because there were significant higher plasma levels for EPA and DHA in the synbiotic, the ldTofa+Syn, and the n3-PUFA groups compared to AIA Ctrl. The lower levels of DHA and AA, and to a lesser degree EPA, in the synbiotic and ldTofa+Syn animals suggest, that these PUFA are increasingly absorbed into the tissue and metabolized in the presence of the synbiotic.

The increased ratio of 18-HEPE+5-HEPE to EPA in the synbiotic and ldTofa+Syn group compared to AIA Ctrl. indicates that with the synbiotic, EPA is more effectively metabolized towards these RvE precursors, hypothesizing that this could also be the case for formation of other SPM. This finding also fits the human data from a previous study that found significantly elevated levels of 18-HEPE and 5-HEPE after supplementation with the synbiotic compared to fish oil with a similar n3-PUFA content (30).

However, the levels of AA-derived PG were substantial in the plasma, as these metabolites function on a more systemic basis. Our data for the cyclooxygenase products PGD_2_, PGE_2_ and TXB_2_ showed significantly reduced levels for animals in the synbiotic and ldTofa+Syn groups compared to n3-PUFA.

These observations align with the results for disease severity parameters, wherein synbiotic and ldTofa+Syn treatment had a beneficial impact, in contrast to n3-PUFA supplementation. Keeping prostaglandins at bay is a relevant factor in RA, as these metabolites were demonstrated to maintain the autoimmune response and inflammation in RA patients (72, 73). Also, they promote leukocyte infiltration, are involved in cartilage degradation and bone resorption, and are important mediators of joint pain regulation (74).

Our data suggests that the supplementation of n3-PUFA alone might not be sufficient for a beneficial effect on disease severity. Instead, we hypothesis that the conversion of n3-PUFA to LM including SPM as well as subsequently altered signal transduction could be crucial determinants of n3-PUFA outcomes.

Based on the correlation between increased EPA derived SPM precursors, reduced PGs, and attenuated disease severity, we hypothesis that the synbiotic may influence the environment of inflammation in the diseased animals, potentially redirecting AA and EPA mediator profiles toward an anti-inflammatory state. However, the exact mechanisms of these processes cannot be deciphered with the analysis method used in this study.

In a retro-perspective view, analyzing SPM not only in the plasma, but also in other relevant compartments of the body such the cell membrane of erythrocytes or the joint fluid could possibly have brought more conclusive results than the analysis of the plasma. Additionally, including all experimental groups of this study in the analysis of the LM would have provided more conclusive mechanistic insights into the effects of the different treatments. These aspects should be addressed in further studies.

### 4.4 Markers of systemic inflammation

ESR and WBC count were analyzed as markers of unspecific inflammation. Changes in ESR are routinely used in the clinic to monitor the treatment efficacy as part of the RA severity score DAS28-4. In contrast to other rat RA models showing a significant increase in ESR upon disease induction, we did not reach a significant difference between healthy and AIA Ctrl. animals, potentially due to the high inter-animal variations (75–77).

The increased WBC found in human RA patients, is normally not positively affected by tofacitinib; some clinical data even indicate a slight increase in WBC in RA patients on tofacitinib in the first month of treatment, with a gradual decline to steady state over the next 4 years (78–80). In line with the human data, tofacitinib did not reduce the AIA-induced leukocytosis in the present study. As the WBC in the ldTofa+Syn group was significantly lower than in the low-dose tofacitinib group, it could be speculated that the synbiotic adds another mode of action to the one of tofacitinib. This WBC-lowering effect could potentially be mediated by effects of the probiotic, as demonstrated for a different bacterium (81), or effects of n3-PUFA or SPM, even though there is no data for the effect of n3-PUFA on WBC or ESR in RA, yet.

The level of IL-6 was demonstrated to correlate with disease activity, as IL-6 contributed to the production of autoantibodies by acting on plasmablasts and CD4^+^ T cell differentiation (44). We revealed increased IL-6 in the AIA Ctrl. group and a trend for reduced IL-6 in animals treated with n3-PUFA, the synbiotic, as well as ldTofa+Syn, while no effect was seen for the two tested tofacitinib concentrations. This is an ambiguous finding, as some rodent studies showed no effect of tofacitinib on blood IL-6 levels (60, 67), but other earlier studies did (82, 83). According to Downty and colleagues, this discrepancy could be related to the time between the last tofacitinib intake and the cytokine measurement, as this drug decays in only 12 h (32). In the present study, sampling was done 24 h after the last dose of treatment, which may have obliviated the effect of tofacitinib, as cytokine levels could have recovered by then. Human studies on the effect of tofacitinib on soluble proteins are limited, but some smaller studies indicated a reduction of IL-6 in the blood of RA patients upon tofacitinib treatment (84, 85). To our knowledge, there are no human studies assessing the effect of n3-PUFA supplementation in RA patients on systemic IL-6. Nevertheless, the trend to reduced IL-6 in animals treated with n3-PUFA, the synbiotic, and ldTofa+Syn is in accordance with work from Morin and colleagues, finding a significant reduction in systemic IL-6 in a model of arthritis upon supplementation with purified EPA (64). A part of the observed effect could also be a consequence of the bacterium, as some probiotic bacteria can modulate systemic IL-6 levels in models of RA (49, 71). Addressing this aspect in more detail could be interesting for future studies.

### 4.5 Markers of joint inflammation

In line with human data, we found increased joint CRP in the AIA Ctrl. group (86). While tofacitinib is well known to reduce serum CRP levels in arthritis models and RA patients (87, 88), data on its effect on local CRP levels is sparse. Our observed reduction of joint CRP with only the high-dose of tofacitinib is in line with data from a similar model (60). As far as we know, there is no data on the effect of n3-PUFA on joint CRP levels in RA. One study with rodents addressed systemic CRP, but found no effect (89). Data on systemic CRP level in RA patients taking n3-PUFA supplements is not conclusive (90). So far, only few rodent models using probiotics addressed CRP levels and found a reduction in systemic CRP (71, 91), and some human trials demonstrated that probiotics diminish systemic CRP levels in RA patients (92). With the strongest reduction found in the ldTofa+Syn group, we propose that tofacitinib and the synbiotic complement each other in the reduction of CRP in the inflamed joints.

A network of various cytokines and cells is involved in the pathogenesis of RA. Local and immigrating immune cells, as well as fibroblast-like synoviocytes (FLS) are responsible for elevated inflammatory cytokines and chemokines in the RA joints (93), promote cartilage degrading enzymes, and enhance osteoclast activity (94).

Many DMARDs target cytokine production and signal transduction directly or indirectly to reduce inflammation and minimize tissue damage in RA patients. Tofacitinib attenuates the JAK1/JAK3-mediated signaling of different cytokines, thereby reducing the expression of different pro-inflammatory genes responsible for triggering and maintaining joint inflammation and tissue damage (94).

In the present study, we did not find an effect of tofacitinib on TNFα, IL-1β, IL-6 or CXCL1 levels in the joints. In contrast to our experience with this model, in this study the level of TNFα in the AIA Ctrl. group was unexpectedly low, making an evaluation of treatment effects difficult. Based on the findings of Downty and colleagues mentioned above, we also speculate that the cytokine levels in the joints may have recovered in the time between the last treatment with tofacitinib and the sampling (32). In line with the importance for sampling time, previous studies demonstrated a local reduction in some cytokines (82, 95), whereas others found only TNFα reduced when tofacitinib was applied at the highest dose of 10 mg/kg (60). Our findings, demonstrating a clear trend in reducing IL-1β and IL-6 and a significant reduction in CXCL1 for the synbiotic, ldTofa+Syn and n3-PUFA group, are in line with previous work showing a reduction in TNFα and/or IL-1β in the joints of animals supplemented with n3-PUFA or specific resolving precursors (61, 96, 97). These results leave us speculating that specific inflammation-mediating n3-PUFA metabolites play a crucial role in keeping cytokine levels in the joints under control for longer than tofacitinib does.

### 4.6 PMN infiltration and NETosis

Neutrophils, the most common cells of the PMN, play a crucial role in the early stage of RA, its disease progression, and perpetuation. The underlying mechanisms include their elevated infiltration into the joint tissue and the synovial cavities, production of ROS, and enhanced formation of NETs (98–100). Citrullinated components of NETs can serve as self-antigens in RA and can initiate the production of anti-citrullinated protein antibodies (ACAP) (99, 101). NETs are formed by ejection of DNA decorated with antimicrobial proteins such as MPO and NE (102–104). In chronic inflammation and RA, cytokines and inflammatory mediators promote this process called NETosis, and MPO and NE can enhance tissue damage as well as inflammatory processes in the joints (101, 105, 106).

In RA patients, markers of NETosis are elevated and many antirheumatic drugs seem to affect neutrophils, even if they don’t directly target these cells (107, 108). Anti-TNFα and anti-IL-6R drugs, for example, reduce markers of NETosis such as MPO and NE significantly (107). In contrast, the effect of tofacitinib on NETosis and PMN has not been extensively investigated. Some studies found moderate effects on absolute blood neutrophil counts, probably by generally decreasing inflammation (108), or by having some effect on the metabolome of neutrophils (109).

In our study, we investigated infiltrating PMN and analyzed the levels of active MPO and NE in the rats’ joints, as an indicator for NETosis of PMN, as they are the vast majority of infiltrating cells in the joint space and are the main source of MPO (99, 107, 110).

Rats treated with 6 mg/kg, but not 3 mg/kg tofacitinib, displayed a strong trend in reducing infiltrating PMN. However, both doses led to a similar significant reduction in active MPO and NE in the joints, suggesting impaired NETosis by reducing infiltrating neutrophils but even more by diminishing the triggers for NETosis.

Current evidence indicates that SPM could reduce PMN transmigration, increase neutrophil clearance, and could be capable of reducing NET formation (111). Accordingly, infiltrating PMN were moderately reduced in the synbiotic group. The most robust trend was seen for ldTofa+Syn, suggesting a potential complementary effect of tofacitinib and the synbiotic on this parameter. Nonetheless, this effect was not seen for MPO and NE. Although the synbiotic led to a moderate reduction, it did not add a benefit on top of tofacitinib.

Notably, n3-PUFA supplementation alone had no effect on active MPO, NE or infiltrating PMN in comparison to the AIA Ctrl. This result was somewhat unexpected, as this treatment reduced the chemoattractant CXCL1. It also stands in slight contrast to two other studies that found reduced neutrophil infiltration with n3-PUFA or resolvins, and to another study that demonstrated reduced levels of active MPO with n3-PUFA (61, 112, 113). However, these discrepancies could be due to the differences in the arthritis models and experimental designs, as well as the different treatment concentrations. To better understand and evaluate these findings regarding PMN and NETosis, additional approaches to investigate their infiltration and function should be included in future experiments.

## 5 Conclusion

Taken together, the results of the present study indicate that the synbiotic ameliorates arthritis disease scores and clinically relevant markers of inflammation, and suggest a complementary effect of this nutritional intervention with the tsDMARD tofacitinib in the treatment of experimental arthritis in rats. This synbiotic approach offers the possibility to serve as new convenient adjuvant therapy option for inflammatory diseases. RA patients could potentially benefit from this new therapeutic approach promoting the resolution of inflammation on top of suppressing immune reactions by current DMARDs. Especially patients who started first-line conventional DMARD therapy could profit, as these drugs alone frequently fail to achieve remission or low disease activity, requiring therapy escalation (3, 114). To assess this possibility in more detail, randomized placebo-controlled studies on patients will be performed.

## Data Availability

The datasets presented in this article are not readily available because of further evaluation of data for patenting processes. Requests to access the datasets should be directed to annette.zehrer@microbiotica.de.
