# Supplementary material for: Enhancing tofacitinib’s therapeutic efficacy in murine arthritis with a synbiotic formulation comprising Bacillus megaterium DSM 32963 and an Omega-3 fatty acid lysine salt
Source: Front Immunol. 2025 May 26;16:1540878. doi: 10.3389/fimmu.2025.1540878 (PMC12146178; doi:10.3389/fimmu.2025.1540878)
Supplement: Supplementary file 1 [file DataSheet1.pdf]

Supplementary Material

1 Supplementary Figures and Tables

1.1 Supplementary Figures

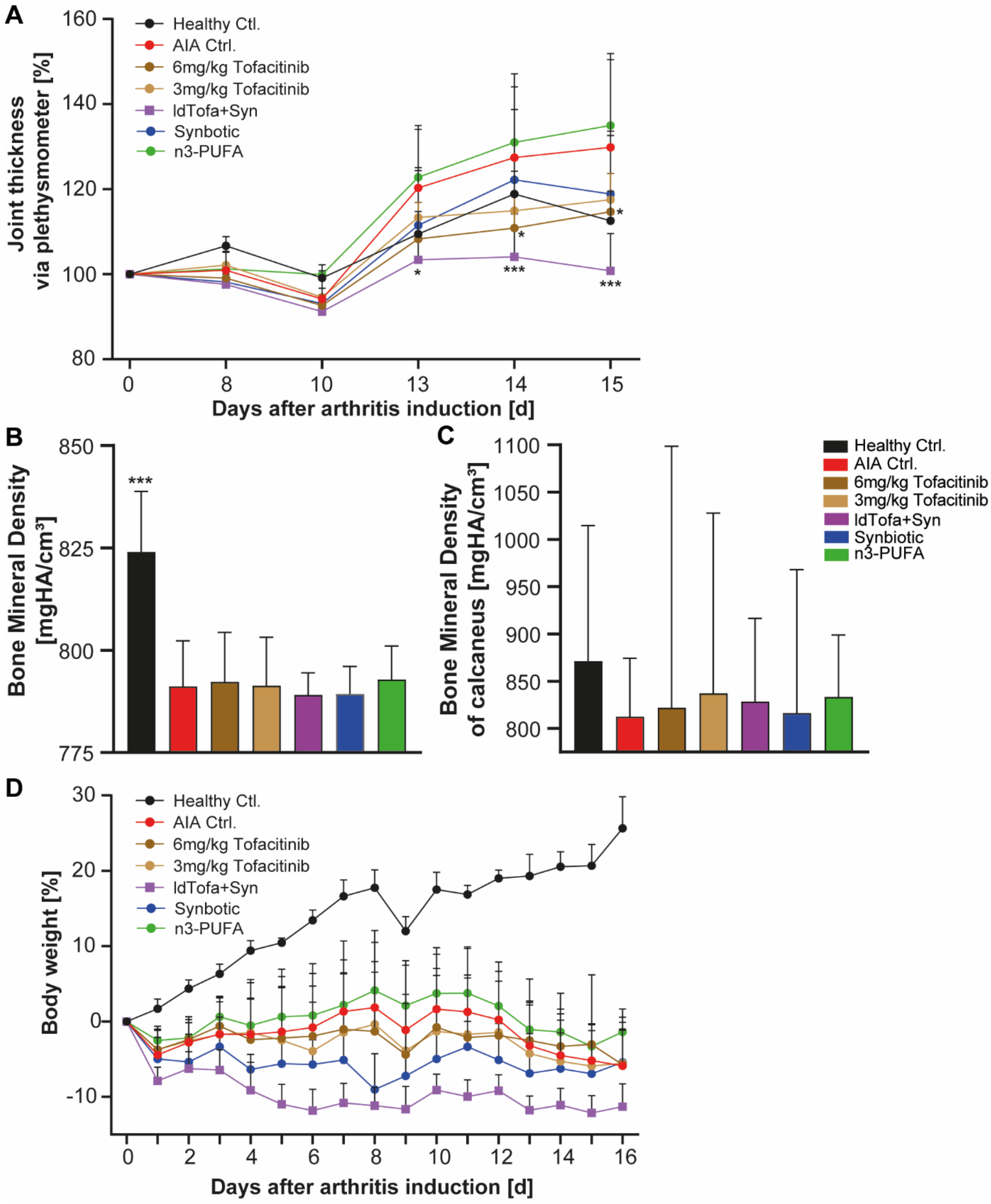

Supplementary Figure 1. (A) Development of joint thickness over time measured via a plethysmometer. **(B)** Bone mineral density (BMD) of the right tarsal joint at day 16**. (C)** BDM of the right calcaneus at day 16. **(D)** Development of body weight over time. n = 2 for (C), otherwise n = 8 / n = 4 healthy Ctrl.; Data represent mean +95% CI; *p < 0.05, **p < 0.01, ***p < 0.001 vs. AIA Ctrl..

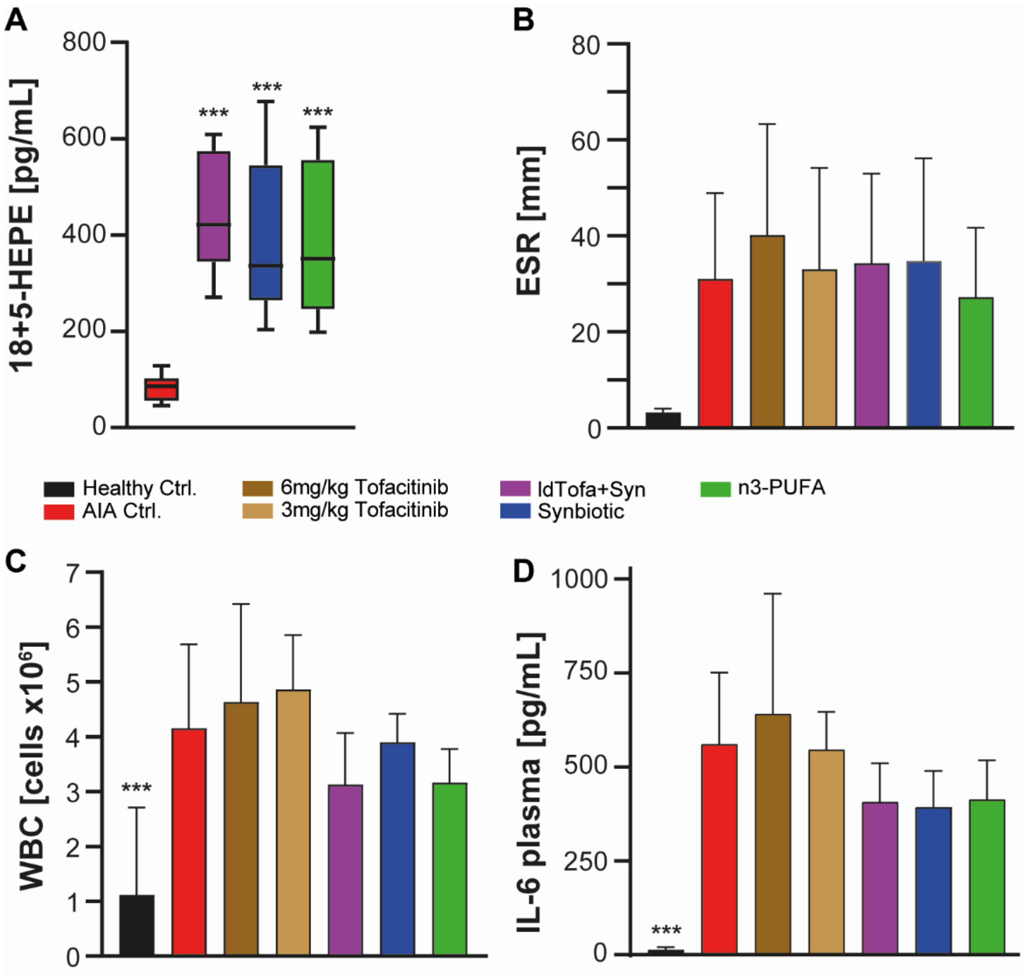

Supplementary Figure 2. (A) Level of 18+5-HEPE in plasma. **(B)** ESR **(C)** WBC **(D)** IL-6 levels in plasma. n = 8 / n = 4 healthy Ctrl.; Data represent mean +95% CI; *p < 0.05, **p < 0.01, ***p < 0.001 vs. AIA Ctrl; Boxplot data represent median, 25th and 75th percentiles, whiskers drawn within the 1.5 IQR value (Tukey method plot), and outliers (empty circles). *p < 0.05, **p < 0.01, ***p < 0.001 vs. AIA Ctrl..
